# Betaine Alleviates High-Fat Diet-Induced Disruption of Hepatic Lipid and Iron Homeostasis in Mice

**DOI:** 10.3390/ijms23116263

**Published:** 2022-06-03

**Authors:** Yanlin Li, Wenduo Jiang, Yue Feng, Lei Wu, Yimin Jia, Ruqian Zhao

**Affiliations:** 1 MOE Joint International Research Laboratory of Animal Health & Food Safety, College of Veterinary Medicine, Nanjing Agricultural University, Nanjing 210095, China; 13592662352@163.com (Y.L.); 18331273006@163.com (W.J.); 2016107034@njau.edu.cn (Y.F.); leiwu@njau.edu.cn (L.W.); jymrobin@hotmail.com (Y.J.); 2 Key Laboratory of Animal Physiology & Biochemistry, College of Veterinary Medicine, Nanjing Agricultural University, Nanjing 210095, China

**Keywords:** betaine, high fat diet, lipogenesis, iron metabolism, DNA methylation

## Abstract

Non-alcoholic fatty liver disease (NAFLD) is characterized by excessive fat deposition in the liver, which is often associated with disrupted iron homeostasis. Betaine has been reported to be hepatoprotective, yet whether and how betaine ameliorates high-fat diet-induced disruption of hepatic lipid and iron homeostasis remains elusive. In this study, mice were fed either standard (CON) or high-fat diet (HFD) for 9 weeks to establish a NAFLD model. Mice raised on HF diet were then assigned randomly to HF and HFB groups, HFB group being supplemented with 1% (*w*/*v*) of betaine in the drinking water for 13 weeks. Betaine supplementation significantly alleviated excessive hepatic lipid deposition and restored hepatic iron content. Betaine partly yet significantly reversed HFD-induced dysregulation of lipogenic genes such as PRARγ and CD36, as well as the iron-metabolic genes including FPN and HAMP that encodes hepcidin. Similar mitigation effects of betaine were observed for BMP2 and BMP6, the up-stream regulators of hepcidin expression. Betaine significantly rectified disrupted expression of methyl transfer gene, including BHMT, GNMT and DNMT1. Moreover, HFD-modified CpG methylation on the promoter of PRARγ and HAMP genes was significantly reversed by betaine supplementation. These results indicate that betaine alleviates HFD-induced disruption of hepatic lipid and iron metabolism, which is associated with modification of CpG methylation on promoter of lipogenic and iron-metabolic genes.

## 1. Introduction

Non-alcoholic fatty liver disease (NAFLD) refers to a range of conditions caused by excessive fat accumulation in the liver, which may progress to nonalcoholic steatohepatitis (NASH), fibrosis, cirrhosis, and even hepatocellular carcinoma. The global prevalence of NAFLD is estimated to be 25% and continues to rise with the obesity epidemic worldwide [1,2]. The main feature of NAFLD is the immoderate accumulation of triglyceride (TG) in the liver, due to disrupted balance of dynamic lipid metabolism [3]. The pathogenesis of NAFLD is often associated with dysregulation of iron homeostasis [4]. However, the relationship between hepatic iron content and NAFLD progression is controversial. High fat feeding for less than 8 weeks leads to higher liver iron deposition [5,6], but this phenomenon disappears after prolonged high fat diet feeding for 10–12 weeks [7,8]. Interestingly, longer high fat diet feeding over 16 weeks decreases the liver iron content [9,10]. Therefore, the hepatic iron deposition in relation to NAFLD progression requires further investigation [11].

The liver is the central regulator for both lipid and iron metabolism. Hepatic lipid content is maintained by a highly coordinated balance between lipid acquisition and lipid disposal. Lipids can be acquired through the uptake of circulating lipids and de novo lipogenesis. Conversely, lipids can be eliminated through fatty acid oxidation and TG export in very low-density lipoproteins (VLDL) particles. Hepatic fat accumulation occurs when lipid acquisition exceeds lipid disposal [3]. The liver also serves as a major reserve for iron storage and acts as a sensor for whole-body iron regulation. The liver takes up transferrin-bound and non-transferrin-bound iron through membrane transport proteins such as transferrin receptors (TFRs), zinc transporter ZIP14, and divalent metal transporter 1 (DMT1), stores iron in a relatively inert state within the iron storage protein, ferritin, and releases iron back into the circulation via ferroportin (FPN) in response to the increased demand for iron. The liver secretes hepcidin, the master regulator of iron homeostasis, which regulates the intestinal absorption of dietary iron and the iron efflux from the liver. Hepatic iron deposition increases when iron uptake and storage exceed the export [11]. Complex interactions between lipid and iron metabolism exist in the liver during the development of NAFLD/NASH [12].

Many genes involved in hepatic lipid and iron metabolism are dysregulated in the progression of NAFLD. Peroxisome proliferator-activated receptor-γ (PPARγ), a multi-functional transcription factor, and the fatty acid translocase CD36, which mediates the hepatic uptake of long-chain fatty acids, are reported to play a causal role in the pathogenesis of NAFLD in mice [13,14,15]. Hepcidin, which is encoded by HAMP gene, prevents cellular iron efflux from the liver by binding to its receptor FPN to induce its internalization and degradation [16]. Iron accumulation in the liver is associated with increased hepcidin production [17] and impaired iron export due to FPN downregulation in NAFLD patients [18], as well as in rats fed high-fat diet (HFD) [6] or high-fructose diet [19].

Increasing evidence indicates the participation of epigenetic mechanisms in the pathophysiology of NAFLD [20,21]. DNA demethylation on PPARγ [22] and PPARα [23] gene promoters was reported in the liver of HFD-induced NAFLD mice and their offspring, respectively. Betaine, a food-derived substance [24], serves as a methyl donor for DNA methylation [25,26,27]. Betaine levels in the blood were inversely associated with the severity of NAFLD in human [28], and many studies suggest a preventive role of betaine in NAFLD [29,30], type 2 diabetes [31] and inflammation [32]. Nevertheless, only two studies reported the efficacy of betaine in the treatment of rat NAFLD models [33,34]. A randomized placebo-controlled trial in human patients reported that betaine did not improve hepatic steatosis but may protect against NAFLD/NASH progression [35]. Betaine attenuates hepatic steatosis of HFD-fed mice by reducing methylation of the microsomal triglyceride transfer protein (MTTP) gene promoter while elevating genomic DNA methylation [36]. However, it remains elusive whether betaine can alleviate disrupted hepatic lipid and iron homeostasis in NAFLD through modification of DNA methylation on genes involved in lipid and iron metabolism.

Therefore, we employed an established NAFLD mouse model to elucidate the effect of betaine on HFD-induced disruption of hepatic lipid and iron homeostasis, and to examine the modification of DNA methylation on affected genes involved in hepatic lipid and iron homeostasis. The results will expand our current knowledge on the efficacy of betaine in treating NAFLD and identify the target genes of betaine action in alleviating NAFLD-associated lipid and iron metabolic disorders in the liver.

## 2. Results

### 2.1. Betaine Alleviates HFD-Induced Disruption of Lipid and Iron Homeostasis

**Figure 1:**
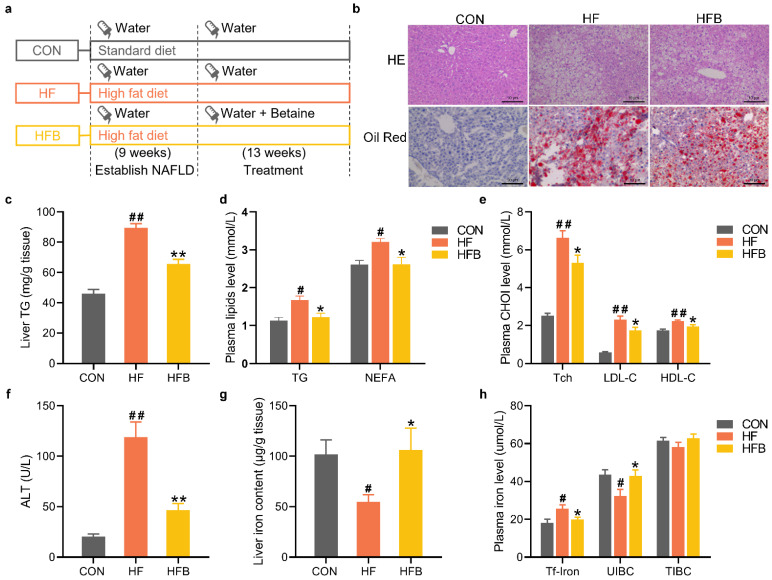
Phenotypic characterization of fatty liver syndrome and liver iron disorder in liver and plasma. (**a**) Flow chart of the experimental design. (**b**) HE staining of hepatic sections (*n* = 3) and ORO staining of hepatic sections (*n* = 3). (**c**) Hepatic content of TG (*n* = 8). (**d**) Plasma level of TG and NEFA (*n* = 8). (**e**) Plasma level of Tch, LDL-C and HDL-C (*n* = 8). (**f**) Plasma level of ALT (*n* = 8). (**g**) Hepatic content of iron (*n* = 8). (**h**) Plasma level of Tf-iron, UIBC and TIBC (*n* = 8). Values are means ± SEM. # *p* < 0.05 and ## *p* < 0.01 compared with the CON group. * *p* < 0.05 and ** *p* < 0.01 compared with the HF group.

Mice were randomly divided into two groups, being fed standard diet (CON) and high-fat diet, respectively, for 9 weeks to establish the NAFLD model. NAFLD mice, while still maintained on high-fat diet, were then divided into HF and HFB groups. Mice in CON and HF group received normal drinking water, while those in HFB group were treated with betaine supplementation in drinking water. The treatment lasted for 13 weeks, and the experimental protocol is shown schematically in Figure 1a. Hepatic pathohistological evaluation showed fatty degeneration with excessive lipid droplets in the liver sections from both HF and HFB groups (Figure 1b). Betaine supplementation partly alleviated the HFD-induced hepatic degeneration, which is supported by smaller vacuoles and less Oil red-straining (Figure 1b), as well as significantly decreased triglyceride (TG) contents (Figure 1c) in the liver. Betaine also significantly reversed HFD-induced increase in plasma levels of TG and NEFA (Figure 1d), total cholesterol (Tch), as well as high-density lipoprotein (HDL) and low-density lipoprotein (LDL) cholesterol (Figure 1e). Accordantly, HFD-induced elevation in plasma alanine transaminase (ALT) activity was significantly rectified in HFB group (Figure 1f), indicating alleviated liver injury.

Meanwhile, betaine supplementation completely mitigated HFD-induced decrease in hepatic iron content (Figure 1g). HFD did not change the total iron binding capacity (TIBC) in the plasma (Figure 1h), yet the plasma concentration of the transferrin-bound iron (Figure 1h) was significantly increased, thus the unsaturated iron-binding capacity (UIBC) was significantly decreased in the plasma of HF mice. Betaine supplementation completely rectified HFD-induced disruption in plasma parameters of iron homeostasis (Figure 1h).

### 2.2. Betaine Reverses HFD-Induced Dysregulation of Pparγ and CD36 in the Liver

**Figure 2:**
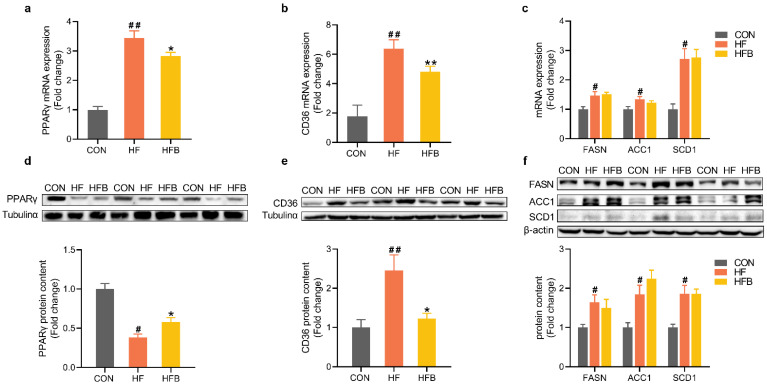
Hepatic genes expression involved in lipid metabolism. (**a**) Hepatic mRNA expression of PPARγ (*n* = 6). (**b**) Hepatic mRNA expression of CD36 (*n* = 6). (**c**) Hepatic mRNA expression of ACC1, FASN and SCD1 (*n* = 6). (**d**) Protein content of PPARγ in the liver (*n* = 6). (**e**) Protein content of CD36 in the liver (*n* = 6). (**f**) Protein content of ACC1, FASN and SCD1 in the liver (*n* = 6). Values are means ± SEM. # *p* < 0.05 and ## *p* < 0.01 compared with the CON group. * *p* < 0.05 and ** *p* < 0.01 compared with the HF group.

Hepatic expression of many lipids metabolic genes was modified by HFD at the level of both mRNA and protein (Figure 2). At the level of mRNA, PPARγ (Figure 2a), CD36, and lipogenic genes such as FASN, ACC1 and SCD1 (Figure 2c) were significantly up-regulated in the liver of HFD-fed mice, as compared with the CON group. Betaine supplementation partly but significantly reversed HFD-induced increase in hepatic mRNA expression of PPARγ (Figure 2a) and CD36 (Figure 2b) but had no effects on the hepatic mRNA abundance of lipogenic genes, FASN, ACC1 or SCD1 (Figure 2c).

At the level of protein, opposite changes were observed for PPARγ (Figure 2d) which was significantly decreased in HF group, and partly yet significantly restored in HFB group. In consistent with their mRNA abundances, hepatic protein content of CD36 (Figure 2e) and lipogenic enzymes (Figure 2f) was significantly increased in HF group. Betaine completely restored CD36 protein content in the liver but had no effects on the hepatic protein content of lipogenic enzymes, FASN, ACC1 or SCD1 (Figure 2f).

### 2.3. Betaine Mitigates HFD-Induced Disruption of Iron-Metabolic Gene Expression

**Figure 3:**
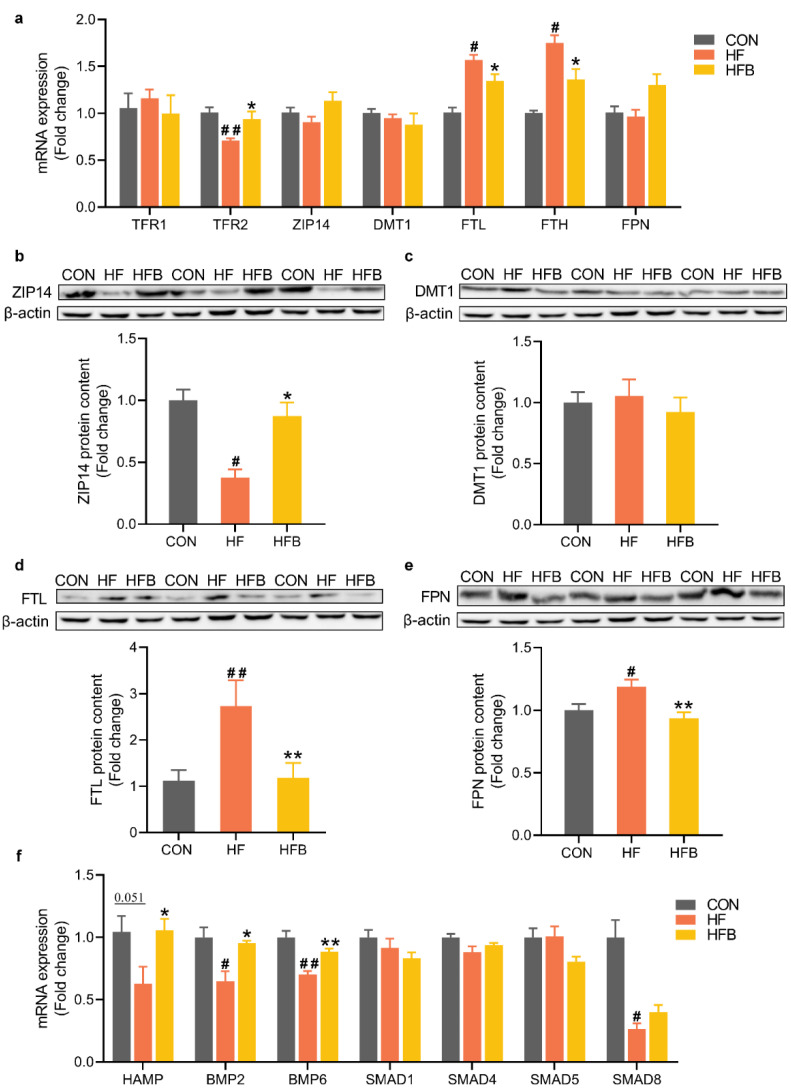
Hepatic genes expression involved in iron metabolism. (**a**) Hepatic mRNA expression of iron metabolism (*n* = 6). (**b**) Protein content of ZIP14 in the liver (*n* = 6). (**c**) Protein content of DMT1 in the liver (*n* = 6). (**d**) Protein content of FTL in the liver (*n* = 6). (**e**) Protein content of FPN in the liver (*n* = 6). (**f**) Hepatic mRNA expression of HAMP and the upstream regulators (*n* = 6). Values are means ± SEM. # *p* < 0.05 and ## *p* < 0.01 compared with the CON group. * *p* < 0.05 and ** *p* < 0.01 compared with the HF group.

At the level of mRNA, transferrin receptor TFR2 was significantly downregulated, while ferritin light chain (FTL) and heavy chain (FTH) were significantly up-regulated in HF group (Figure 3a). Betaine supplementation significantly reversed these HFD-induced dysregulations (Figure 3a). At the level of protein, zinc transporter ZIP14 (Figure 3b) was significantly decreased, while FTL (Figure 3d) and FPN (Figure 3e) were significantly increased in HF group. All these HFD-induced changes were restored by betaine supplementation (Figure 3b,d,e). Hepcidin, the liver-produced master regulator of iron homeostasis encoded by HAMP gene, was downregulated (*p* = 0.051) at the level of mRNA in HF group, and completely reversed in HFB group (Figure 3f). Similar patterns were observed for the upstream regulators of hepcidin, BMP2/6 and SMAD8, which were significantly downregulated in HF group. Betaine completely reversed HFD-induced downregulation of BMP2 and BMP6, but not SMAD8 (Figure 3f).

### 2.4. Betaine Restores HFD-Induced Dysregulation of Methyl-Transfer Genes

**Figure 4:**
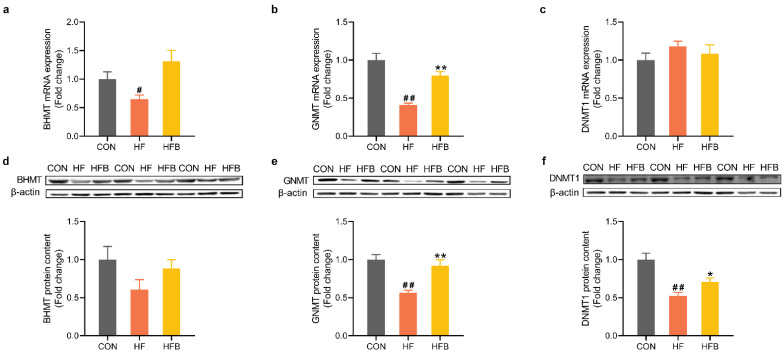
Hepatic genes expression involved in methyl-transfer. (**a**) Hepatic mRNA expression of BHMT (*n* = 6). (**b**) Hepatic mRNA expression of GNMT (*n* = 6). (**c**) Hepatic mRNA expression of DNMT1 (*n* = 6). (**d**) Protein content of BHMT in the liver (*n* = 6). (**e**) Protein content of GNMT in the liver (*n* = 6). (**f**) Protein content of DNMT1 in the liver (*n* = 6). Values are means ± SEM. # *p* < 0.05 and ## *p* < 0.01 compared with the CON group. * *p* < 0.05 and ** *p* < 0.01 compared with the HF group.

Methionine cycle plays a critical role in methyl transfer and thus the epigenetic gene regulation. HFD induced a significant downregulation of glycine N-methyltransferase (GNMT) and betaine-homocysteine methyltransferase (BHMT), at mRNA (Figure 4a,b) and/or protein (Figure 4d,e) levels. Betaine supplementation completely restored hepatic GNMT expression at both mRNA (Figure 4b) and protein (Figure 4e) levels. Hepatic protein content of DNA methyltransferase 1 (DNMT1) was significantly decreased in HF group, and partly yet significantly restored in HFB group (Figure 4f).

### 2.5. Betaine Rectifies HFD-Modified DNA Methylation on Promoter of Affected Genes

**Figure 5:**
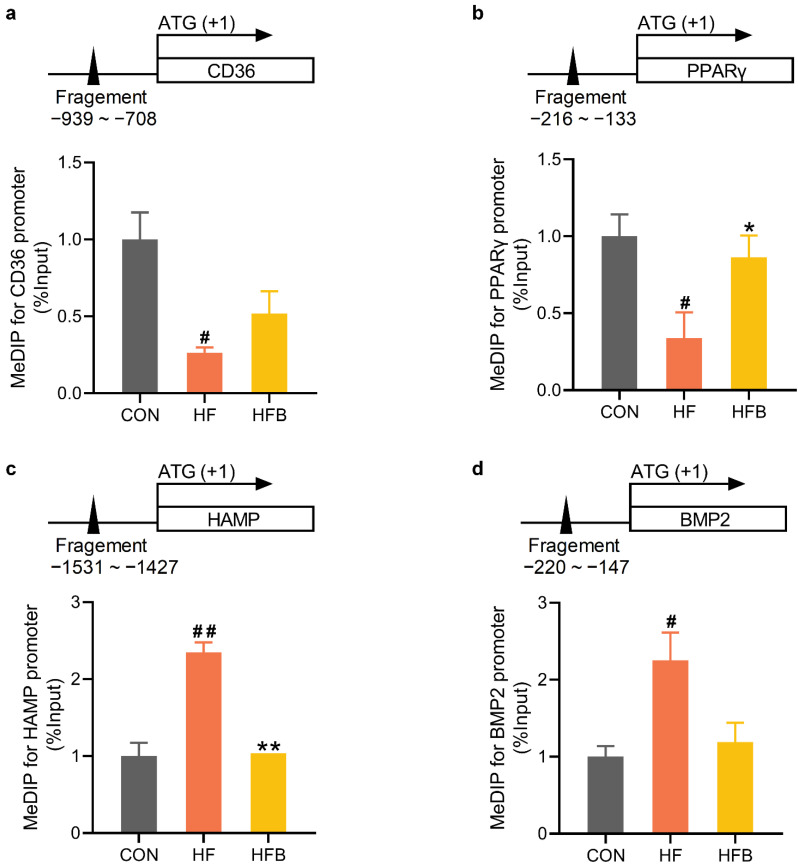
Hepatic methylation level on promoter of affected genes. (**a**) Methylation status on the promoter of CD36 gene (*n* = 3). (**b**) Methylation status on the promoter of PPARγ gene (*n* = 3). (**c**) Methylation status on the promoter of HAMP gene (*n* = 3). (**d**) Methylation status on the promoter of BMP2 gene (*n* = 3). Values are means ± SEM. # *p* < 0.05 and ## *p* < 0.01 compared with the CON group. * *p* < 0.05 and ** *p* < 0.01 compared with the HF group.

The promoter regions of CD36 (Figure 5a) and PPARγ (Figure 5b) genes were significantly hypomethylated, which coincided with higher abundance of their mRNAs detected in the liver of HF mice. HAMP (Figure 5c) and BMP2 (Figure 5d) gene promoters were significantly hypermethylated, corresponding to diminished mRNA expression of these two genes in the liver of HF mice. HFD-induced modification of DNA methylation on PPARγ and HAMP gene promoters was significantly rectified by betaine supplementation, which is associated with reversed mRNA expression in the liver of HFB mice. Betaine partially restored on the methylation status of CD36 (*p* = 0.117, Figure 5a) and BMP2 (*p* = 0.124, Figure 5d) gene promoters, leading to reversed expression of these genes in the liver of HFB mice.

## 3. Discussion

In this study, betaine supplementation in drinking water significantly alleviated the excessive lipid deposition and restored disrupted iron homeostasis in the liver of HFD-fed mice. The results provide new evidence to support the therapeutical effect of betaine on established NAFLD mouse model. We identified PPARγ/CD36 and HAMP/FPN as target genes of betaine action on lipid and iron metabolism, respectively. Moreover, the mitigation effects of betaine on HFD-induced disruption of hepatic lipid and iron homeostasis are associated with the restoration of promoter DNA methylation status on PPARγ and HAMP gene.

Hepatic iron deposition appears to vary depending on the stage or severity of NAFLD progression. Higher liver iron deposition was reported in animal models subjected to HFD feeding for less than 8 weeks [5,6], while no changes in liver iron content were reported in animals fed HFD for 10–12 weeks [7,8]. More prolonged HFD feeding over 16 weeks led to decreased liver iron content [9,10]. The reason for such stage-dependent changes in liver iron content is widely unknown, probably due to complex interactions among different cell types in the liver during NAFLD progression. In this study, mice in HF group were fed HFD for 22 weeks, 9 weeks for establishing NAFLD and 13 weeks serving as a control for betaine treatment. In line with the previous reports, we detected decreased hepatic iron content in HFD-fed mice in association with increased hepatic lipid accumulation. Liver iron content is negatively correlated with the serum iron concentration in HFD-fed mice [37]. The same is true in this study as mice in HF group exhibited higher plasma concentration of transferrin-bound iron and lower iron content in the liver.

The mechanisms underlying the excessive hepatic lipid deposition in NAFLD also differ in different animal models. Some studies reported enhanced uptake of circulating lipids and de novo lipogenesis [15,38,39], while others reported impaired fatty acid oxidation and TG export [40,41,42]. Numerous studies reported contribution of hepatic up-regulation of CD36 to NAFLD pathogenesis [13,14,15]. In support of these findings, we found that the hepatic expression of CD36 was up regulated in HF group and reversed in HFB group, at both mRNA and protein levels. CD36 is a transcriptional target of PPARγ in promoting hepatic steatosis [43]. In this study, hepatic expression of PPARγ mRNA showed the same pattern as CD36 in response to HFD feeding and betaine supplementation. However, reversed pattern of PPARγ protein was observed, being decreased in HF group, and restored in HFB group. Many studies showed decreased hepatic PPARγ protein content in mice fed HFD and restored PPARγ protein content in mitigation group [44,45,46]. PPARs are ligand-activated transcription factors, which regulate energy homeostasis via modulation of different downstream target genes in a tissue- and cell-specific manner [47]. PPARγ, which is highly expressed in adipose tissue, promotes insulin sensitivity by increasing adipose lipid storage and reducing adipose fatty acid influx into the liver [48], whereas its role in the liver remains unclear. The uncoupling of mRNA abundance and protein content of PPARγ in the liver implies a post-transcriptional regulation of PPARγ expression. Hepatic expression of lipogenic genes, including FASN, ACC1 and SCD1, was significantly enhanced by HFD, but was not restored by betaine supplementation. Therefore, betaine attenuates HFD-induced dysfunction in hepatic lipid metabolism via targeting PPARγ/CD36 pathway in the liver. The target genes of betaine action appear to vary in different animal models with different experimental design. In a short-term (28-d) study [49], oral gavage of betaine at 400 mg/kg protected rats from HFD-induced hepatic lipid accumulation via up-regulating the fatty acid oxidation genes PPARα and CPT1.

Hepatic iron content is determined by a dynamic balance among iron uptake, storage, and output. In this study, decreased hepatic iron deposition was restored by betaine supplementation. In screening the genes involved in betaine action, we found that ZIP14, which mediates iron uptake, FTL, the light chain of the iron-storage ferritin, and FPN that is responsible for iron efflux, are all dysregulated by HFD and reversed by betaine. HFD-induced decrease in hepatic iron content is associated with decreased ZIP14 and increased FPN expression in the liver, suggesting reduced iron uptake and enhanced iron efflux. Our findings agree with previous reports that HFD increases FPN expression [9], and reduces ZIP14 expression [50] in the liver. Betaine restores hepatic iron content by targeting ZIP14 and FPN that are involved in iron uptake and efflux, respectively. FPN is regulated by hepatocytes-derived hepcidin encoded by HAMP gene. Hepcidin binds to FPN to induce its internalization and degradation [16], thus preventing iron efflux. HAMP and its upstream BMP-SMAD pathway [51] appear to be the targets of betaine action, because HFD-induced suppression of HAMP and BMP2/6 expression was restored by betaine supplementation.

Betaine is involved in the methionine cycle for the conversion of homocysteine to methionine, which is further converted to SAM, a universal methyl donor for numerous methylation reactions, including DNA methylation catalyzed by DNA methyltransferases (DNMTs). Numerous studies indicate the role of betaine in methyl transfer and DNA methylation [26,52]. In this study, GNMT and DNMT1 was decreased by HFD and restored by betaine, which implies the participation of DNA methylation in the therapeutical action of betaine on HFD-induced NAFLD. Locus-specific methylation analysis revealed HFD-induced modifications of DNA methylation on CD36, PPARγ, HAMP and BMP2 gene promoters. Interestingly, CD36 and PPARγ gene promoters were hypomethylated, while HAMP and BMP2 gene promoters were hypermethylated, which corresponds, respectively, to increasedCD36/PPARγ mRNA and decreased HAMP/BMP2 mRNA in the liver of HFD-fed mice. Betaine completely or partially restored HFD-induced modifications of DNA methylation on the promoter of these genes, and thereby reversed the pattern of their mRNA expression. Betaine was found to alleviate HFD-induced NAFLD in mice by reducing MTTP promoter methylation while elevating global DNA methylation [36]. We did not determine the alterations of global DNA methylation in this study, but enhanced DNMT1 expression in HFB group implies similar changes. The mechanisms underlying the gene- and locus-specific modification of DNA methylation induced by HFD or betaine remain unknown, presumably involving complex interaction of DNA methylation machinery with other co-regulators of gene expression.

## 4. Materials and Methods

### 4.1. Animals and Treatment

Twenty-four 4-week-old SPF C57BL/6 mice were obtained from the Center of Comparative Medicine at Yangzhou University and raised at the Experimental Animal Center of Nanjing Agricultural University under the condition of 22 ± 0.5 °C at 50 ± 5% humidity, with a 12-h light-dark cycle. After 1 week of acclimatization, the mice were randomly divided into two groups, being fed a control diet with 10% of energy from fat (CON, *n* = 8) and a high-fat diet (HFD) with 60% of energy from fat (*n* = 16), respectively, for 9 weeks. The nutritional composition of the experimental diets is shown in Table 1. Then the HFD-fed mice were subsequently assigned to HF and HFB subgroups while remaining on HFD for another 13 weeks. Mice in HF group were provided with normal drinking water, while those in HFB group received drinking water supplemented with 1% (*w*/*v*) betaine (98% purity, B2629, Sigma-Aldrich, Saint Louis, MO, USA). Mice in CON group were fed the control diet with normal drinking water throughout the experiment. At the end of the 13th week, the mice were fasted overnight before euthanasia. Blood was collected and plasma samples were prepared and stored at −20 °C before use. Liver samples were harvested and stored at −80 °C. All procedures with animals were approved by the Animal Ethics Committee of Nanjing Agricultural University. The sampling procedures followed the ‘Guidelines on Ethical Treatment of Experimental Animals’ (2006) no.398 set by the Ministry of Science and Technology, China.

### 4.2. Determination of Plasma Biochemical Parameters

Plasma alanine aminotransferase (ALT) activity (H001), nonesterified fatty acid (NEFA, OD444), triglyceride (TG, H201), total cholesterol (Tch, H202), low density lipoprotein cholesterol (LDL-C, H207), high density lipoprotein cholesterol (HDL-C, H203), transferrin-bound iron (Tf-iron, H309W) and total iron binding capacity (TIBC) (H465) were measured with an automatic biochemical analyzer (Hitachi 7020, HITACHI, Tokyo, Japan) using respective commercial assay kits purchased from Ningbo Medical System Biotechnology Co., Ltd. (Ningbo, China).

### 4.3. Hepatic Histological Evaluation

Fresh liver tissue was fixed with 4% paraformaldehyde, embedded in paraffin, and then cut into sections of 4 μm in thickness and stained with hematoxylin and eosin (H&E). To visualize the hepatic fat droplets, fresh frozen liver samples were embedded in optimal cutting temperature (OCT) compound and sliced into 8 μm sections. The frozen sections were stained with oil red O (Sigma Aldrich, Saint Louis, MO, USA) for 30 min, counter-stained with H&E for 30 s, then mounted in neutral resin. The slides were observed by using an optical light microscope (Olympus-BX53, Tokyo, Japan).

### 4.4. Determination of Hepatic Triglyceride Content

Hepatic triglyceride (TG) content was measured by a commercial assay kit (A110-1-1) purchased from Nanjing Jiancheng Bioengineering Institute (Nanjing, China), following the manufacturer’s instructions.

### 4.5. Total RNA Isolation and Real-Time PCR

Total RNA was isolated from liver samples with TRIzol reagent (Invitrogen, Carlsbad, CA, USA) and reverse-transcribed according to the manufacturer’s protocol (R223-01, Vazyme, Nanjing, China). Diluted cDNA (2 μL, 1:25) was used for real-time PCR on Biosystems QuantStudio 6 Flex Real-Time PCR System (Applied Biosystems, Foster City, CA, USA). All primers (Table 2) were synthesized by GenScript Biotech (Nanjing, China). PPIA was chosen as a reference gene. The 2^−ΔΔCT^ method was used to analyze the real-time PCR data.

### 4.6. Western Blot Analysis

Total protein was extracted from liver samples as previously described. Protein concentration was measured with the Pierce BCA Protein Assay kit (Thermo Fisher, Waltham, MA, USA) according to the manufacturer’s instruction. Western blot analysis of ACC1 (4190, Cell Signaling Technology, USA, diluted 1:1000), FASN (3189, Cell Signaling Technology, USA, diluted 1:1000), SCD1 (2438S, Cell Signaling Technology, USA, diluted 1:1000), PPARγ (WL01800, Wanleibio, China, diluted 1:500), CD36 (BS7861, Bioworld, China, diluted 1:1000), FTL (A18051, Abclonal, China, diluted 1:1000), FPN (DF13561, Affinity, China, diluted 1:1000), ZIP14 (ab123988, Abcam, USA, diluted 1:1000) and DMT1 (ab55733, Abcam, USA, diluted 1:1000) were carried out following the manufacturer’s instructions. Tubulin-α (BS1699, Bioworld, China, diluted 1:5000) or β-Actin (AC026, ABclonal, China, diluted 1:100,000) was used as internal control.

### 4.7. Methylated DNA Immunoprecipitation (MeDIP) Analysis

MeDIP was carried out as previously described [53]. In brief, 1 µg genomic DNA purified from liver was fragmented to a mean size of 300 bp by sonication, then heat denatured and immunoprecipitated with 5-mC antibody (ab10805, Abcam, UK) overnight at 4 °C. The immunoprecipitated DNA captured by pretreated protein A/G agarose (sc-2003, Santa Cruz Biotechnology) was recovered with proteinase K digestion followed by phenol-chloroform-isoamyl alcohol (25:24:1) purification. The recovered DNA fractions were diluted 1:50 and used to amplify the proximal promoter sequences of mouse CD36, PPARγ, HAMP, and BMP2 genes by real-time PCR with specific primers (Table 2).

### 4.8. Statistical Analysis

Differences among groups were analyzed by *t*-test using SPSS 20.0 software (SPSS Inc., Chicago, IL, USA). Data are expressed as means ± SEM. The differences were considered statistically significant when *p* < 0.05.

## 5. Conclusions

We provide new evidence that betaine alleviates the HFD-induced disruption of hepatic lipid and iron homeostasis in mice, probably via the regulation of lipid- and iron-metabolic genes associated with the modification of promoter DNA methylation. It is noted, however, that here we show the collective responses of gene regulation to HFD or betaine in the liver regardless of hepatic cell heterogeneity. As different cell types, including hepatocytes, Kupffer cells and hepatic stellate cells, perform distinct functions in lipid and iron metabolism in different stages of NAFLD progression, more in-depth investigations are needed to understand the cell-specific gene regulation and their interactions underlying the therapeutical efficacy of betaine in the treatment of NAFLD.

## Figures and Tables

**Table 1:** Composition and nutrient content of the experimental diets.

	CON		HF	
Energizing material	gm%	Kcal%	gm%	Kcal%
Protein	19.2	20	26.2	20
Carbohydrate	67.3	70	26.3	20
Fat	4.3	10	34.9	60
Total		100		100
	3.85		5.24	
Ingredient	gm	Kcal	gm	Kcal
Casein, 80 Mesh	200	800	200	800
l-cystine	3	12	3	12
Corn starch	315	1260	0	0
Maltodextrin 10	35	140	125	500
Sucrose	350	1400	68.8	275.2
Cellulose	50	0	50	0
Soybean Oil	25	255	25	255
Lard	20	180	245	2205
Mineral mix S10026	10	0	10	0
Dicalcium Phosphate	13	0	13	0
Calcium Carbonate	5.5	0	5.5	0
Potassium Citrate,1 H_2_0	16.5	0	16.5	0
Vitamin Mix V10001	10	40	10	40
Choline Bitartrate	2	0	2	0
Pigment	0.05	0	0.05	0
Total	1055.05	4057	773.85	4057

**Table 2:** Nucleotide sequences of primers.

Target Genes	Primer Sequences (5′to 3′)	
qPCR PPARγ	F: CTTGCAGTGGGGATGTCTCA	R: CCTCGCCTTTGCTTTGGT
CD36	F: TTGATGTGCAAAATCCACAGG	R: TGTGTTGTCCTCAGCGTCCT
ACC1	F: GGAGATGTACGCTGACCGAG	R: TACCCGACGCATGGTTTTCA
FASN	F: GGCCCCTCTGTTAATTGGCT	R: GGATCTCAGGGTTGGGGTTG
SCD1	F: CCTCCGGAAATGAACGAGAGA	R: ATCCCGAAGAGGCAGGTGTA
TFR1	F: TCGGAGAAACTGGACAGCAC	R: ATCACGCCAGACTTTGCTGA
TFR2	F: GGTCTATTCCAGAGAGCGCA	R: CGACGTAGCCCAGTAGGAAG
ZIP14	F: AGAAGGTCATTGTGGGCTCG	R: AGTGAAGGAAGCACCGATGG
DMT1	F: AGCTGTCATCATGCCACACA	R: AGACTTCAACCACCTGCTCG
FTL	F: ATTTCGACCGCGATGATGTG	R: CATGGCGTCTGGGGTTTTAC
FTH	F: GCCATCAACCGCCAGATCAA	R: AAGATTCGGCCACCTCGTTG
FPN	F: GAGATCACAACCGCCAGAGA	R: CACATCCGATCTCCCCAAGT
HAMP	F: CTTTGCACGGGGAAGAAAGC	R: TGCAGATGGGGAAGTTGGTG
BMP2	F: TAGTGTTGCTGCTTCCCCAG	R: CTCCACGGCTTCTTCGTGAT
BMP6	F: TCTCCCCACATCAACGACAC	R: AAACTCCCCACCACACAGTC
SMAD1	F: GCCTCTGGAATGCTGTGAGT	R: GAACTGAGCCAGAAGGCTGT
SMAD4	F: TTCAAGCTGCCCTGTTGTGA	R: GACCTTTATATACGCGCTTGG
SMAD5	F: TGTTGGGCTGGAAACAAGGT	R: GTGACACACTTGCTTGGCTG
SMAD8	F: TCAACACTCAGACTTCCGGC	R: TTGAGAGAAGCCCAAGGCAG
PPIA	F: GACTGAGTGGTTGGATGG	R: TGATCTTCTTGCTGGTCTT
MeDIP		
PPARγ	F: AGAGAGAGAGATGAAAAGCACATC	R: GTGTCCCTCAGACCGATGTC
CD36	F: GCAGGCACAATGTAAGACCAA	R: TGTCATCAACCTCAGCCATTC
HAMP	F: TCCCCAAGAGATTGGCTCAC	R: AGGGGCCAACAGGAGTACTT
BMP2	F: GTGGACTCTGGATTTGCCCTA	R: TGAGTGTGAAGCCGACCCC

## Data Availability

Not applicable.
